# “Got to build that trust”: the perspectives and experiences of Aboriginal health staff on maternal oral health

**DOI:** 10.1186/s12939-020-01301-5

**Published:** 2020-10-23

**Authors:** Ariana C. Kong, Mariana S. Sousa, Lucie Ramjan, Michelle Dickson, Joanne Goulding, Kylie Gwynne, Folau Talbot, Nathan Jones, Ravi Srinivas, Ajesh George

**Affiliations:** 1 Centre for Oral Health Outcomes and Research Translation (COHORT), School of Nursing and Midwifery, Western Sydney University/South Western Sydney Local Health District / Ingham Institute for Applied Medical Research, Liverpool, 2170 NSW Australia; 2 IMPACCT - Improving Palliative, Aged and Chronic Care through Clinical Research and Translation, Faculty of Health, University of Technology Sydney, Broadway, 2007 NSW Australia; 3 Translational Health Research Institute, Campbelltown, 2560 NSW Australia; 4 Sydney School of Public Health, Faculty of Medicine and Health, University of Sydney, Camperdown, 2006 NSW Australia; 5 Primary and Community Services, South Western Sydney Local Health District, Liverpool, 2170 NSW Australia; 6 Faculty of Medicine and Health Sciences, Macquarie University, Macquarie Park, 2109 NSW Australia; 7 Faculty of Medicine and Health, University of Sydney, Camperdown, 2006 NSW Australia; 8 Aboriginal Health Unit, South Western Sydney Local Health District, Liverpool, 2170 NSW Australia; 9 Oral Health Services, South Western Sydney Local Health District, Liverpool, 2170 NSW Australia; 10 School of Dentistry, Faculty of Medicine and Health, University of Sydney, Camperdown, 2006 NSW Australia

**Keywords:** Aboriginal, Dental, Pregnancy, Qualitative, Yarning, Model of care

## Abstract

**Background:**

In Australia, models of care have been developed to train antenatal care providers to promote oral health among pregnant women. However, these models are underpinned by Western values of maternity care that do not consider the cultural needs of Aboriginal and Torres Strait Islander women. This study aimed to explore the perceptions and experiences of Aboriginal health staff towards oral health care during pregnancy. It is part of a larger program of research to develop a new, culturally safe model of oral health care for Aboriginal women during pregnancy.

**Methods:**

A descriptive qualitative methodology informed the study. Focus groups were convened to yarn with Aboriginal Health Workers, Family Partnership Workers and Aboriginal management staff at two antenatal health services in Sydney, Australia.

**Results:**

A total of 14 people participated in the focus groups. There were four themes that were constructed. These focused on Aboriginal Health Workers and Family Partnership Workers identifying their role in promoting maternal oral health, where adequate training is provided and where trust has been developed with clients. Yet, because the Aboriginal health staff work in a system fundamentally driven by the legacy of colonisation, it has significantly contributed to the systemic barriers Aboriginal pregnant women continue to face in accessing health services, including dental care. The participants recommended that a priority dental referral pathway, that supported continuity of care, could provide increased accessibility to dental care.

**Conclusions:**

The Aboriginal health staff identified the potential role of Aboriginal Health Workers and Family Partnership Workers promoting oral health among Aboriginal pregnant women. To develop an effective oral health model of care among Aboriginal women during pregnancy, there is the need for training of Aboriginal Health Workers and Family Partnership Workers in oral health. Including Aboriginal staff at every stage of a dental referral pathway could reduce the fear of accessing mainstream health institutions and also promote continuity of care. Although broader oral health policies still need to be changed, this model could mitigate some of the barriers between Aboriginal women and both dental care providers and healthcare systems.

**Supplementary information:**

The online version contains supplementary material available at 10.1186/s12939-020-01301-5.

## Background

Oral health is important during pregnancy. An estimated 50–74% of women have periodontitis (gum disease) during pregnancy [1, 2], which is associated with an increased risk of pre-eclampsia [3], pre-term birth and low birth weight [4]. A mother’s oral health and dental practices are also associated with the child developing dental decay across the lifespan [5]. Pain or functional limitations resulting from oral health problems can also have a considerable impact on the mother’s quality of life during pregnancy and daily activities of children like eating and sleeping [6, 7]. Moreover, untreated oral health problems can be extremely costly [8].

The impact of poor oral health is exacerbated in socially disadvantaged families particularly those from culturally and linguistically diverse communities, as well as Indigenous communities (Additional file 1). In Australia, early childhood decay affects 40% of all children and the prevalence is much higher among the Aboriginal and Torres Strait Islander communities (hereafter referred to as Aboriginal, Additional file 1) [9]. Further, improving the maternal and infant oral health outcomes of Aboriginal communities continue to be a focus of the Australian government’s national strategy to close the gap [10–12]. However, accessing health services can be both traumatic and stressful for some Aboriginal Australian women during pregnancy [13]. These feelings may arise because of the longstanding impact of colonisation (Additional file 1), leading to the loss of land, culture and language [14, 15]. Many Aboriginal Australians experience regular and ongoing personal and systemic racism [12, 16]. These social determinants have an association with wellbeing and self-efficacy [17], which is an individual’s confidence in their ability to enact a desired behaviour [18]. Among Aboriginal mothers, having a high level of self-efficacy is an important factor in engaging in oral health behaviours such as accessing the dentist [19, 20]. With a collective history of oppression and dispossession, sense of self-efficacy among Aboriginal people may be readily depleted with negative experiences of attempted behavioural change [18].

Another determinant impacting dental behaviours of Indigenous women are the health policies that influence access to affordable dental care [21]. In Australia, 67% of health care is publicly-funded through the universal health care insurance scheme, Medicare [22]. The Medicare Medical Benefits Schedule provides support for a range of health care-related expenses [22]. Medicare-funded dental care, however, is more restricted to certain populations and is guided by the state or territory’s health department [23]. In New South Wales (NSW), for example, adult residents need to be listed on a government-issued concession card to access the public dental service. Although dental services are also provided specifically for Aboriginal people through some Aboriginal community-controlled health services (ACCHSs) in NSW, the eligibility criteria may vary between ACCHSs [24].

Further, despite oral health recommendations for women during pregnancy [25, 26], oral health tends to be neglected by many antenatal care providers such as general practitioners (GPs), obstetricians/gynaecologists and midwives [27]. A model of care that includes training for midwives has been successfully developed and implemented in Australia to promote oral care for women during pregnancy [28]. While this model is effective in improving the competency of midwives to promote oral health as well as the oral health outcomes of expectant mothers [28–30], it was developed for health care services that uphold Western values of care. Consequently, the approach of current antenatal oral health models of care may not be culturally safe for many Aboriginal women.

Successful maternity care models among Aboriginal Australian women have included antenatal care providers who identify as Aboriginal, are connected to the Aboriginal community, and can provide culturally safe care with an Indigenous worldview [31]. These maternity care models may involve a partnership or team comprising of midwives, Child and Family Health Nurses, Aboriginal ‘senior women’, Aboriginal Health Workers (AHWs), Aboriginal Maternal and Infant Care workers or Family Partnership Workers (FPWs) [31–33]. Thus, to meet the cultural needs of Aboriginal communities, existing oral health programs for antenatal care providers need to be adapted for services that already provide culturally safe care to Aboriginal clients.

Aboriginal antenatal care providers, such as AHWs, have the potential to promote oral health care during pregnancy [34]. Within Australia, AHWs already promote oral health among other populations including children [34]. The aim of this study, therefore, was to understand the experiences and perceptions of Aboriginal antenatal care providers, specifically AHWs and FPWs, and Aboriginal management staff, towards oral health care for women during pregnancy. This study is part of a larger program of research, informed by a participatory action research (PAR) approach, to develop a culturally safe model of care to meet the oral health needs of Aboriginal pregnant women and new mothers.

## Methods

### Methodology

This study was underpinned by a qualitative descriptive methodology that employed yarning as a method [35–37]. Yarning involves conversing to share stories and exchange Aboriginal knowledge in a way that prioritises Aboriginal ways of knowing, being and doing [35–37]. It focuses on establishing and building respect, reciprocity and trust [37], and is a credible and rigorous method of research [37, 38].

### Conceptualisation of study

Prior to the study, the lead author (AK) yarned with the Aboriginal health staff, who identified that antenatal oral health was identified as an area of importance. The Aboriginal staff also identified the desired outcomes for the study (that is, a culturally safe model of care) and described how they wanted to be involved in the study (through a focus group and periodic meetings for brainstorming and key decision making with various aspects of the model of care). To inform the model of care, the Aboriginal staff specified that yarning in focus groups with Aboriginal health staff was the most appropriate method of data collection, followed by interviews with Aboriginal pregnant women. These yarns informed the topic areas that could be explored during focus groups. The findings from the interviews with the Aboriginal pregnant women will be published elsewhere.

### Ensuring a cultural lens

The initial yarns were important to cultivate trust between the Aboriginal staff and the lead author (AK), who identifies as a non-Indigenous Australian woman and convened the focus groups. In addition to the AHWs and FPWs, the study team included non-Indigenous (LR, MSS, AG, KG) and Aboriginal researchers (MD, JG, FT) who were involved in the analysis and interpretation of the findings. Both Aboriginal (NJ, BR) and non-Indigenous people (RS) with experience in delivering health services, assisted with decision making throughout the study.

### Ethical considerations

Ethical approval for this study was granted from the South Western Sydney Local Health District (2019/ETH09963) and the Aboriginal Health & Medical Research Council (1438/18). Reciprocal approval was also granted from Western Sydney University (RH13086).

### Context

This study was conducted in the Greater Western Sydney region in NSW, Australia. The study included staff from two antenatal health service programs designed for Aboriginal pregnant women and new mothers. These programs have an outreach model which involve AHWs or FPWs alongside nurses visiting clients to provide clinical antenatal support.

### Sampling

Purposive sampling was used to recruit management staff and AHWs and FPWs involved in two different antenatal outreach programs. There were no exclusion criteria.

### Data collection

The focus groups were conducted through yarning. Yarning involves a relaxed conversation and discussion where external researchers learn from Aboriginal peoples about their lived experiences, stories, thoughts and feelings [37]. It is different from traditional focus groups as Aboriginal peoples are the custodians of knowledge. Since yarning is part of Aboriginal and Torres Strait Islander ontology, epistemology and axiology [38], it creates a safe space for Aboriginal peoples to exchange knowledge [38]. Although in this study, non-Indigenous researchers convened ‘focus group’ discussions (the term ‘focus group’ will be used for consistency), they became yarning circles where the Aboriginal health staff exchanged knowledge about their own perspectives and personal views of Aboriginal women’s experiences through shared stories in an Aboriginal way. This exchange, using yarning, changed the dynamic of the focus group so that the non-Indigenous researchers would learn from the Aboriginal health staff [38]. Including the perspectives of the Aboriginal health staff on the experiences of Aboriginal women was intentional to compare and triangulate the findings with the perceptions of Aboriginal women during pregnancy. A semi-structured approach to the focus groups was adopted, using five key issues to guide the focus group yarning:

- Previous experiences (struggles/successes) caring for Aboriginal and/or Torres Strait Islander pregnant women (tease out the nature of their relationship)
- Knowledge about antenatal oral health (problems faced, priorities, practices, trends from both theirs and clients’ perspectives)
- Potential education, assessment and referral (challenges and facilitators)
- Education and training (their needs/how they envision it)
- Other comments/questions

Since responses to the key issues prompted spontaneous questions or discussion, the focus group structure was flexible. Yarning provided the Aboriginal staff the freedom to linger on certain areas, to explore unanticipated topics and the opportunity for priorities to be identified by the Aboriginal staff, rather than by the non-Indigenous researchers, ensuring that all views were canvassed and respected. This approach facilitated a dynamic exchange where Aboriginal knowledge could be taught and shared by the Aboriginal staff to the non-Indigenous researchers, building trust and reciprocity.

A total of three focus groups were conducted in private rooms at the community health centres where the Aboriginal staff were based. Prior to providing consent, AK reiterated verbally (supported by the participant information sheet) that the participants were in a safe, confidential space. The staff were not obliged to participate, nor were there consequences for non-participation, and they could withdraw consent at any time.

Two focus groups were conducted at one service to allow all staff to attend. A third focus group with the FPWs was conducted at the other service. The first focus group was convened by two of the study authors (AK and LR), where LR (a qualitative researcher) also wrote field notes. The subsequent two focus groups were facilitated only by AK, who wrote field notes after each focus group. All focus groups were audio recorded. The recordings were transcribed by a professional service and checked for accuracy by AK.

### Participant demographics

Fourteen people participated in the three focus groups, including 7 AHWs, 2 Aboriginal management staff (who were not AHWs), and 5 FPWs. The FPWs’ position descriptions were similar to AHWs, in that they raised cultural awareness within their teams to ensure culturally safe service delivery but did not necessarily require the same qualification. All participants were female, with an age range of 22 to 50 years. The highest educational qualification attained by staff included Year 12 (*n* = 1), vocational education (*n* = 8) or a university qualification (*n* = 3). Three staff, who already had vocational education, were also working towards a university qualification. Two people chose not to disclose this information. Years of experience working as an AHW ranged from three to 10 years, whereas the FPWs’ years of experience were between 2 weeks to 8 years.

### Analysis and interpretation

An inductive thematic analysis [39] was employed. All participants were assigned pseudonyms to ensure confidentiality. AK read and re-read the transcripts, listened to the audio recordings, read accompanying field notes and wrote additional memos to ensure adequate immersion in the data. AK initially coded the transcripts inductively using NVivo software. The initial codes were generated verbatim or in summary about an issue relating to the research aim. AK clustered similar codes together, generating initial categories. AK revisited these categories a second and third time for further understanding, and then combined the categories into themes across all focus groups. These themes were reviewed by another non-Indigenous researcher with experience in qualitative research (MSS) and by an Aboriginal researcher (FT). After agreement between AK, MSS and FT, AK convened with the AHWs and FPWs separately to yarn about the themes. This allowed for participants to check, engage and further contribute to the interpretation of the data, and ensure rigour [40]. All participants were invited for a follow-up discussion about the analysis, however only half of the participants (*n* = 7) were available due to unforeseen changes with client scheduling. This discussion refined the concepts underlying each theme and the language used to define the themes.

## Results

Four main themes emerged from the focus groups (Table 1) relating to the Aboriginal staff’s perspectives and experiences of maternal oral health care. All findings around the scope and future design of an antenatal oral health program will be published elsewhere.

**Table 1:** Focus group themes and sub-themes

***Theme***	***Sub-Theme***
More oral health knowledge and training to meet the local community’s needs	• Understanding Aboriginal ways of doing health service provision
	• Current oral health training, knowledge and practices
Trust builds empowered relationships	• Building trust
	• Supporting women to make choices
Colonisation and intergenerational trauma: systemic barriers	• External barriers to accessing dental services
	• Feelings of ‘shame’: fear, anxiety and judgement
Systems that provide continuity of care	• Working in two worlds
	• Need for a priority dental referral pathway

### Theme 1: more oral health knowledge and training to meet the local community’s needs

The Aboriginal health staff recognised the importance of oral health during pregnancy and agreed that they could provide oral health education to pregnant Aboriginal women and mothers as part of their role. The participants discussed appropriate Aboriginal ways of doing in health services and highlighted the need for training in antenatal oral health.

#### Understanding Aboriginal ways of doing health service provision

Participants identified that any oral health training should be integrated into an existing antenatal program. Two of the staff identified a potential role for Elders (see glossary, Additional file 1) to pass on knowledge about healthy oral health practices.

*I think you could build it into the [antenatal] program, though.* (Sharon, management staff)

*I think, um, culturally we always go to our Elders for guidance so I think for the Elders to, um, have an opportunity to filter down ideas, guidance, support - that's an appropriate way for us. So I guess keeping in with that, um, you know, speak…and having that yarn and consultation with them.* (Louise, AHW)

*If we say something, then their grandma says something, they're not going to go say what we say – they’re going to listen to their [Elders]* (Teigan, FPW)

#### Current oral health training, knowledge and practices

One FPW had acquired oral health knowledge through formal training. All other participants across both services identified the need for a formal oral health training program.

*I think I’ve just learnt it [oral health] over the training that I’ve done, like Certificate III and IV in Aboriginal and Torres Strait Islander Primary Health Care…then over my lifetime…I know I’ve got a thing about teeth.* (Melissa, FPW)

*Yeah, I think informal as well. I mean, we did do little in-services. We do do in-services on dental, so it could be some formal as well…I would be up for it [formal training]* (Emily, AHW)

Across both services, the AHWs and FPWs already had some knowledge of the effect of pregnancy on a woman’s oral health and vice-versa and understood the importance of a healthy diet for the mother’s and baby’s teeth. Several participants already encouraged women to see the dentist. The FPWs also handed out dental products to families.

*So I know that during pregnancy, women's oral health can be exasperated from pregnancy. You know, that can cause wobbly teeth, it can cause decay to happen quicker, so it exasperates all of the symptoms, so I do know that. Um, it can cause headaches. It can cause other health concerns. It can stop them eating. It can give them anxiety. All kinds of different things* (Emily, AHW)

*If the client hasn't seen a dentist in a while, we usually ask them when was their last dental check-up.* (Melissa, FPW)

*So when we're talking about any good foods, we talk about the type of food you do that are better for your teeth rather than the sugary ones and the soft drinks and all that…About if you're having a lot of soft drinks which are high caffeine and high sugar that's going through to bub.* (Louise, AHW)

*I know with some of our clients, that we've gone out and some of the content we've - it's touched on the oral health, we've given, like in the gift packs, we've given out the toothpaste and toothbrush.* (Melissa, FPW)

### Theme 2: trust builds empowered relationships

The AHWs and FPWs from all the focus groups identified that promotion of oral health care needed to be provided in the context of relationships where trust was established with their clients. Building trust would ensure that they could give culturally safe support to Aboriginal pregnant women and mothers during the antenatal period.

#### Building trust

Trust was discussed as an imperative to understand their clients’ needs and priorities. Building trust required time, empathy and sharing personal experiences (yarning).

*Trust. Got to build that trust.* (Melissa, FPW)

*It's totally up to them and what they want. We tend to find that if we just sit there and have a yarn with them rather than push them. We find a lot of services do try and push, you known like, to tell the girls what to do. We don't do that: and we find we get better outcomes when we don't do that.* (Karina, FPW)

*But it's also too with that rapport building is that those yarns that you're having with your clients aren't about this is what you're doing, it's about you giving them your experience as well. So, you know, it's like, I've done this too.* (Tess, AHW)

Reflecting on the importance of trust, both groups found that Aboriginal women tended to be more receptive to contact from AHWs or FPWs compared to nurses.

*Often, at times, when they're in crisis and they don't answer the phone calls to the nurses, all it takes is one phone call from us and then we're back on board with them…when we contact them they're usually pretty honest with us about what's going on with them.* (Sarah, AHW)

*We basically just support the nurses. Already questions that the client might have. Sometimes they …direct the questions at us rather than the nurse. We just bounce off one and another and just support, you know, what we’re delivering* (Karina, FPW)

#### Supporting women to make choices

Following the discussion about the importance of building trust, the AHWs spoke about offering choices and providing support by ensuring that the clients’ needs were addressed. Regardless of whether this support was psychosocial, practical or both, the staff spoke about how this approach addressed some of the barriers that affected women’s access to services.

*Yes, and I actually just tend to ask, do you have somebody you can go with or do you feel okay doing this, um, or are you all right to make the call? Or if you haven't got credit, do you need to use my work phone or do you want to wait until you've got credit? So always giving options or if they've got ideas, well what do you think? So they'll let us know if they can't do that.* (Louise, AHW)

*it depends on where the mum is at, I guess. I'll say it that way… Yeah, their ability to access, whether they're comfortable calling, because I'll call for some clients… We do provide transport if we need to as well.* (Emily, AHW)

When asked a question about the nature of the relationships the AHWs and FPWs had with the clients, some of the participants described themselves as being the connectors and interpreters for many clients.

*I guess we're that connector, we're the connecter with a system that is different traditionally to what some of our systems would be or would look like. So we help break down the barriers of, um, an institution which has historically been, um, one that's had a negative attachment to it from past policies and history.* (Louise, AHW)

*So it's kind of like - I think of us as…friendly – not a friend. Um, we look after their cultural stuff, you know, to help support them with culture? Um, we're the link between mainstream and Aboriginal people and Aboriginal culture stuff. We're kind of like interpreters as well? Because a lot of clinical stuff is a lot of jargon, so we, um, will explain it in a different way. We advocate – [emphasised] a lot.* (Emily, AHW)

### Theme 3: colonisation & intergenerational trauma: systemic barriers

The long-term effects of colonisation and intergenerational trauma (Additional file 1), which affected clients’ desire and ability to engage with services and institutions, were discussed in all focus groups. The Aboriginal staff spoke about the barriers for clients to access dental services. These barriers include cost of dental treatment, transport, dentists refusing to treat pregnant women, long-waiting time for an appointment, ineligibility to access ACCHS (Aboriginal community controlled health service) dental services, and systemic racism. The participants also identified that ‘shame’ (see glossary, Additional file 1) which accompanied feelings of fear, anxiety and being judged during a dental appointment, were factors that could affect an Aboriginal woman’s desire to visit the dentist.

#### External barriers to accessing dental services

The AHWs and FPWs estimated how many of their clients (out of 10) would have dental problems during pregnancy. One participant said *“I’ve had two”* (Melissa, FPW), whereas others agreed that the number was closer to *“six to eight”* (Rachel, AHW) out of 10. Two participants agreed that only *“one to two”* (Melody, AHW) actually end up attending a dental appointment.

Cost, transport, and dentists who refused to treat pregnant women were cited by AHWs as some reasons for poor uptake of dental services.

*They're thinking they have to go private and they don't have money.* (Sarah, AHW)

*It's quite hard - especially if you don't drive and you have to catch public transport.* (Tess, AHW)

*When I was pregnant with my last one, my tooth was actually bad up the back, and I went to the dentist and they refused to touch it because I was pregnant* (Rachel, AHW)

Some FPW staff also shared personal experiences or knowledge of the long waiting lists to access public and ACCHS dental services.

*I went privately. I was like I need to get this out. It was killing me. I've had a wait list over at [ACCHS dental service], because I went to [ACCHS dental service] …but then the wait list to get my tooth removed was like a year?* (Ellie, FPW)

*If you don't want to pay, like the waiting list for the one at [public dental service], for example. [exasperated sigh]* (Teigan, FPW)

The participants spoke about the need for a Health Care Card (concession card) to access public dental services; however, not all Aboriginal women qualified for this card if they were on a higher income. Furthermore, for Aboriginal women who were on a higher income, money was prioritised elsewhere.

*Because I earn over the threshold, you don't get the free dental.* (Emily, AHW)

*the Health Care card is the biggest issue. If they're still working while they're antenatal - they can't go and access [the public dental service] because they're still getting paid…They just can't financially afford to go to a dentist, but then on a higher income - because of choices of buying a home which is what we want to do…* (Louise, AHW)

One person spoke about how negative experiences with government institutions created fear and became a deterrent for families to access government services. This staff member explained that even if these experiences were with one institution, the fear created a spill-over effect to any government institution.

*That [institutions] goes hand in hand. [with racism]* (Sally, AHW)

*There are so many complexities sometimes that it's really difficult for families to engage with Centrelink to chase that. They might have previous debt. They might have a child that's come, that's left their care, and they're backwards and forwards and it's all too hard to go into Centrelink and negotiate in that space. So that whole fear of institutional contact is...So it’s the fear of going in and having to deal with that entity, that institution, that’s why Aboriginal families prefer that outreach contact.* (Jennifer, management staff)

Five participants explained that since ACCHSs provide free dental services for Aboriginal Australians, some non-Aboriginal people identified as being Aboriginal to access these services.

*Dental's one of them. That's why they're* [people who didn’t identify previously as Aboriginal] *identifying, so they can get free access to it.* (Karina, FPW)

To address this, some ACCHS require ‘confirmation papers’ (Confirmation of Aboriginality) (see glossary, Additional file 1). Participants identified confirmation papers as a barrier for many clients, especially if the client was disconnected with their family because of policies leading to the Stolen Generations.

*That's why it's harder to get the confirmation now, because people were just going and using names and getting their confirmation, where now you need to go to these meetings and it is harder… But then it's harder for people that are from Stolen Generations and don't have - and are disconnected with their family. It's just so - it's just all a big mess.* (Ellie, FPW)

The participants described the process of acquiring Confirmation of Aboriginality to be a long process.

*That is pretty much - there's nearly a 12-month waiting list. So you fill in your application form, hand that in, then … once it's your turn they'll send you a letter and say this is the day and time that you need to present in front of the board – um, the board will ask you a couple of questions, and then it goes from there. So whether they accept it or not… More information, exactly. Come back or go back to where your family is known.* (Melody, AHW)

### Feelings of ‘shame’: fear, anxiety and judgement

The Aboriginal staff extensively discussed the shame, anxiety and fear associated with oral health and accessing dental services within the community. This anxiety and fear resulted from personal experiences or stories heard from within the community.

*My dad’s tooth just fell out…like the whole thing. He put it in the bin. I said, why did you do that? [Unclear]. I said, why didn't you take it to the dentist, and they can put it back in? He's like, nup. My nan yells at him every day, like rips him up. She says, you can't get jobs with teeth like that you need to go and fix your teeth.* (Ellie, FPW)

*There's also just dental in general, the horror stories…and shame* (Rachel, AHW)

*Sometimes it's the elders, they instil the fear, I've got to say, because my grandmother wouldn't go into hospital. Never went to a hospital. Some of my immediate relatives could be in there dying, she won't go to a hospital. She wouldn't go and see a doctor. She wouldn't go to a dentist. God, no, she never went to a dentist. Even though my nan had false teeth, she never went to a dentist in her life.* (Jennifer, management staff)

The Aboriginal staff also discussed that feelings of shame arose from being embarrassed or from the fear of being judged.

*Some people that we spoke to did this. [covers mouth with hand] Covered their mouth when they were talking to us.* (Jennifer, management staff)

*But also, I wouldn't initiate this story about how my parents didn't give me a toothbrush or do that, because I wouldn't want people to judge my parents. I'm sharing because it's safe.* (Sharon, management staff)

*I've got false teeth. Mine are through domestic violence. You know I mean? You've got to be careful on ‘em lines too. Like, I don't mind talking about it. I'm strong enough to talk about it. But there's some that don't - you know what I mean, admit to that.* (Karina, FPW)

The participants discussed the effect of past policies of assimilation that allowed for the removal of children, enforced English as the only language that could be spoken, and policed cultural practices and activities. Some participants mentioned that as a result, knowledge, language and culture were not passed down to younger generations, including the passing down of traditional dietary and dental health knowledge and practices.

*Back in the day you weren’t allowed to [talk to anyone]… Doesn’t matter if you were Stolen or not. Yeah, you just weren’t allowed to. It was part of the white law at the moment. You know what I mean?* (Karina, FPW)

*There's certain stuff, yeah, that they chew on and stuff like that, but no one’s ever really passed that down.* (Karina, FPW)

*Just living on bush tucker and nothing out there to hurt your teeth.* (Melody, AHW)

*Yeah, and this is how it went off-track and the introduction of a Western diet, and when you think about why people choose the bottle over the breast and, you know, what they put in, it's because of what's going on…and you need to capture that from Aboriginal people. Um, some of that you can see how some people do know here, and how it's okay to regain that knowledge, because the same way why other knowledge hasn't been passed down, this is, you know, the same reason. So that, I think, is really important.* (Sharon, management staff)

### Theme 4: systems that provide continuity of care

#### Working in two worlds

The participants discussed how they found themselves balancing their professional roles while also maintaining their cultural responsibilities within the community. The Aboriginal staff spoke about having a role in both ‘worlds’, suggesting that the services’ policies were not always culturally safe.

*Like, um, we obviously work under policies and guidelines, um - we're always competing with - what is culturally safe and appropriate versus policies that we've got [to] work under. So we're always adapting to make it work in regards to what we're allowed and what we know within ourselves as Aboriginal people what is actually appropriate to do within the homes.* (Louise, AHW)

One AHW shared an example where the existing workplace policies meant that Aboriginal clients had no option to access culturally safe and affordable dental services:

*Well, I have a client that's just relocated from Melbourne. She's Aboriginal, no confirmation papers, she's not on the pension card, Health Care card, and her teeth are pretty much not there. What access does she have? Any kind of money that she has - she's got five - six children now. Very young mum, 23…. It’s a brick wall. That’s just an example.* (Sally, AHW)

#### Need for a priority dental referral pathway

All focus groups stressed the need for priority dental referral pathways that would provide free dental check-ups for all Aboriginal pregnant women and for women who were pregnant with an Aboriginal child. This was considered an important preventative initiative for the community.

*…in an ideal scenario we can get them in and get them streamlined to have that check-up then as a preventative measure for when, as you just said, pregnancy and everything.* (Sarah, AHW)

*Maybe that could be something, an escalated pathway so people in the program can make sure that within that - we get them checked within a...* (Sharon, management staff)…*Certain timeframe, like a KPI [key performance indicator]* (Jennifer, management staff)

*But if we offered it as something that was offered to everyone across the board, it wouldn't be so confronting… You know, so if it was something that was offered to everyone [all pregnant women with Aboriginal babies]* (Sally, AHW)

Some participants suggested that there should be an initial dental appointment available to clients to raise awareness about existing oral health problems and subsequently discuss their potential risk.

*Because then you go to a dentist and you find out. Because I wouldn't go unless I had an issue; then I would go* (Ellie, FPW)

*So I don't know how that would fit but in my ideal world once she's pregnant I think she should be able to receive some treatment, whether that be an examination and fillings or what not, what they can do during the pregnancy* (Sharon, management staff)

One management staff recommended that all mothers with Aboriginal babies needed pathways to a range of public and private services, including ACCHSs.

*So I think if you attach the model to your program, that could have several pathways. One into the AMS [Aboriginal medical service], because we do outreach there and we do different pathways and, you know, there's no wait for any Aboriginal child, so why can't we have that for our unborn child and mothers? And then you've got the voucher system, where if you're needing services [the AMS] can't provide, you can get a voucher…into private dental.* (Sharon, management staff)

It was important that non-Aboriginal mothers of Aboriginal babies could also access culturally safe services because they were still considered part of the community.

*So when we look at a holistic thing so that non-Aboriginal mum that's pregnant within our Aboriginal community, even though she's not seen as Aboriginal she's still seen as a part of our community* (Louise, AHW)

Another suggestion was issuing all women with a concession card during their pregnancy to ensure that dental services were accessible to all women. Some participants were cautious about the potential for further discrimination if only Aboriginal women received a priority referral.

*It could work from once - like from my perspective then all - well not just Aboriginal women, all women who are pregnant, there's a guideline that they have to book in before 20 weeks gestation. So, everyone is under that umbrella, who knows whether they've been booked in or not, and that's including Centrelink and everyone else…so every antenatal mum who has booked in why can't they be issued with a healthcare card for the duration for when she's pregnant? Why can't that be an open healthcare card that's given to all regardless of how much you earn and things like that?* (Tess, AHW)

*But how that’s rolled out, there needs to be some sort of consultation around it to be mindful about stigma that’s already attached, you know, prejudice that’s already attached…my worry would be how it’s done, done in the best way* (Louise, AHW)

## Discussion

This study sought to understand the perspectives and experiences of AHWs, FPWs and Aboriginal management staff to inform a model of care that could address the oral health needs of Aboriginal pregnant women and new mothers within the community. The Aboriginal health staff acknowledged that promoting oral health among Aboriginal pregnant women could be a part of the role of AHWs and FPWs, which is a first in Australia. The components of a new model would need to include capacity building AHWs and FPWs in oral health promotion using training that follows Aboriginal ways of doing and implement strategies that escalate the cultural safety of dental services. More broadly, policies need to be negotiated with governments and ACCHSs to better address the oral health needs of Aboriginal women.

To provide enhanced oral health care for Aboriginal women during pregnancy, the Aboriginal health staff identified that the AHWs and FPWs could play a role if training is provided. Although some participants had knowledge about maternal oral health, this was learned informally and did not provide the staff formal qualifications. This is unsurprising as a recent review [34] found that no antenatal oral health training programs have been developed and evaluated for Indigenous health workers globally. Furthermore, there are currently no national perinatal oral health workforce strategies in Australia. However, oral health training for AHWs and FPWs, who already provide antenatal services, could further redress the impact of colonisation by providing an opportunity for Aboriginal women to receive oral health knowledge in ways that are culturally safe. Some studies with Australian Aboriginal communities revealed that the effectiveness of a health program is linked to how well the program adopts the community’s cultural practices and knowledge, and directly involves the community [41–44]. In the context of PAR which is informing the larger program of research, the team (including the Aboriginal health staff) will need to design the training program, based on the suggested content and delivery, using the insights gained through these focus groups. Potential solutions to improve culturally safe care within the public dental service, such as involving Aboriginal dental staff at all points of care, will also need to be integrated in a model of care.

Yet, promoting oral health among Aboriginal pregnant women needs to be through Aboriginal ways of doing. Building trust with clients was important because it was the way that the AHWs and FPWs could effectively support clients to make informed decisions, and take action, about their health. The priority to build trust changes the dynamic in the health provider-client relationship to one that is comparable to a partnership. As discussed by Karina and Tess, trust is built through yarning, a traditional method of knowledge exchange in Aboriginal cultures [45]. Gaining and maintaining this trust through yarning could be a potential factor to building sense of self-efficacy [18]. Listening to the shared experiences of AHWs and FPWs may provide some clients the psychosocial support necessary to seek dental care. This type of support was an important factor identified by Kong et al. [21] in maintaining oral hygiene practices among Indigenous women. Yet, trust with an individual care provider has its limitations when situated within the broader healthcare system [46].

The long-term effects of colonisation and intergenerational trauma are two main factors that have, and continue to, generate distrust and fear of health systems among Aboriginal peoples. The Aboriginal staff shared specific experiences where people refused to access mainstream health institutions. The government policy of assimilation, where Aboriginal children were forcibly removed from their families, has led to a significant loss in identity, culture and family connection, resulting in intergenerational trauma [14, 16]. This in turn has resulted in a pervasive fear and distrust among Aboriginal peoples when accessing and engaging with mainstream health institutions. The participants’ discussion around shame and fear of being judged for having poor oral health highlighted a systemic problem among health care services failing to deliver culturally safe care [47, 48]. Some mainstream health care services have attempted to address the cross-cultural gap by employing AHWs to engage with Aboriginal clients [49]. In a similar model, public dental services could redress some of the effects of colonisation and mitigate some of the fear by having an Aboriginal person as the first point-of-contact over the phone to manage dental appointments. Employing at least one Aboriginal person at each dental clinic would also facilitate culturally safe continuity of care. Thus, there is the need for both the system and its health care providers to focus on providing care that aligns with Aboriginal ways of doing.

The prerequisite of Confirmation of Aboriginality to some ACCHS dental services reveal the cycle created by historical colonising policies, which initially excluded Aboriginal peoples from receiving culturally safe health care, and inadvertently continues to exclude Aboriginal peoples from culturally-specific health care services. Some Aboriginal women experienced increased difficulty obtaining Confirmation of Aboriginality if they had relocated from where they were known by community or could not reconnect with community because of the past policy of assimilation. Given that some non-Aboriginal people fabricate being Aboriginal to access affordable dental services, this highlights issues with the cost of dental services more broadly. Difficulties with affording regular private dental treatment is a challenge for both Aboriginal and non-Aboriginal Australian families [50]. These complex issues should be discussed with government policy makers and ACCHSs to explore relaxing stringent eligibility criteria to dental services during pregnancy, and better address the needs of Aboriginal families.

The barriers to dental care demonstrate the need for provision of culturally safe and inclusive continuity of care. However, Aboriginal care providers may need to negotiate with the policies of a non-Indigenous organisation and draw on inherent Aboriginal ways of knowing and being to provide this care [51, 52]. To practically support Aboriginal staff who navigate these two worlds, a range of referral pathways into public, private and ACCHS dental services need to be implemented so that all women pregnant with Aboriginal babies can access dental care, irrespective of Aboriginal status. In the UK, free dental treatment is offered to all women during pregnancy and up to 12 months after delivery [53]. In one Australian study, promoting oral health and subsidising the cost of dental check-ups significantly increased access the dental service during pregnancy [30]. Although subsidising dental check-ups may be costly initially, offering pregnancy dental checks may be more economical as it promotes long term preventive dental care for both the mother and the child [54].

Despite the strengths, there were some limitations in this study. The AHWs and FPWs who participated worked in an urban area; therefore, the perspectives of AHWs and FPWs working in regional or remote areas were not identified. As every Aboriginal community is unique, the perspectives shared are not intended to be representative of other AHWs or FPWs. Moreover, the AHWs or FPWs were not trained and employed to collect or analyse the data due to the heavy workload capacity of AHWs and FPWs and restrictions in funding.

## Conclusions

The perspectives of Aboriginal health staff, who work within antenatal services, has provided valuable insight into the complexities and potential solutions around oral health among Aboriginal pregnant women and new mothers. Some of the challenges experienced by Aboriginal women highlight the need for changes in policy around accessing dental services. The AHWs and FPWs identified their role in promoting oral health as part of a new model of care. However, formal oral health training for AHWs and FPWs is still needed. To increase cultural safety, a proposed model could include Aboriginal staff who are present at every stage of a dental referral pathway to facilitate continuity of care. Future research and development of this model of care should be developed with the AHWs and FPWs using a PAR approach to ensure it is culturally safe and addresses the maternal oral health needs of Aboriginal women.

## Supplementary information

Additional file 1. (DOCX 14 kb).

## Data Availability

The data used and/or analysed for this study are available from the corresponding author on reasonable request.

## Abbreviations

AHWs: Aboriginal Health Workers
FPWs: Family Partnership Workers
ACCHSs: Aboriginal community controlled health services
AMS: Aboriginal medical service
PAR: Participatory Action Research
