# Supplementary material for: “Got to build that trust”: the perspectives and experiences of Aboriginal health staff on maternal oral health
Source: Int J Equity Health. 2020 Oct 23;19:187. doi: 10.1186/s12939-020-01301-5 (PMC7585174; doi:10.1186/s12939-020-01301-5)
Supplement: Supplementary file 1 — (DOCX 14 kb). [file 12939_2020_1301_MOESM1_ESM.docx]

Appendix 1

| **Glossary: Terminology and definitions** | |
| --- | --- |
| ***Terminology*** | ***Definition*** |
| **Indigenous peoples** | Referring collectively to all distinctive peoples worldwide who identify as being descendants of the first peoples or custodians of a specific region. |
| **Aboriginal and/or Torres Strait Islander peoples/Australians** | Referring specifically to the multitude of distinct peoples and nations who were the first peoples and custodians to own the land in Australia. |
| **Colonisation** | During the 1700s, British government recognised Australia to be *terra nullius* (a Latin word referring to a *land belonging to nobody*), providing a legal basis to unilaterally dispossess the land from the existing landowners. Colonisation refers to the process in which the British government began to settle and occupy the land. |
| **Elders** | Elders in Aboriginal communities, not to be confused with elderly people, are highly respected and recognised by the community as persons gifted with knowledge and wisdom to provide leadership, education, spiritual guidance and healing for the community.[43, 44] |
| **Stolen Generations** | The Australian government’s policy of assimilation (1937-1973) aimed to remove all other cultures and languages practised by Aboriginal and Torres Strait Islander peoples. This policy led to the forcible removal of many Aboriginal and Torres Strait Islander children from their parents to assimilate into Western society. The children who were removed from their families are known as the ‘Stolen Generations’. |
| **Intergenerational trauma** | Trauma refers to a person’s response to an overwhelming event that leaves a person unable to cope with the experience. British colonisation and the subsequent Australian policies dispossessed many Aboriginal and Torres Strait Islander Australians from culture, land, language, kinship group and identity. For some people, the trauma from colonisation has been passed down from older to younger generations in the form of mental health problems, violence, parenting practices and behavioural problems. |
| **Confirmation of Aboriginality** | Certain organisations may request people to provide a ‘Confirmation of Aboriginality’ to access Aboriginal-specific services or programs. To acquire these documents will require the applicant to meet the criteria of being and identifying as Aboriginal or Torres Strait Islander descent and being accepted as such in the community in which a person lives or previously resided. Applications for the confirmation are typically reviewed by a registered Aboriginal community organisation, which will also issue the documentation. |
| **Shame** | While the English word ‘shame’ is typically used to refer to feelings of humiliation caused by doing something wrong, the AHWs in this study and Aboriginal peoples more broadly, appropriate this word to encompass feelings associated with a fear of disapproval or judgement, shyness, embarrassment, a lack of respect and breaches of cultural and social norms. |
| **ACCHS (Aboriginal community-controlled health service) /AMS (Aboriginal medical service)** | A non-government primary health care service that is initiated by, based in, and provides health services and programs for the local Aboriginal community. Health services and programs that are delivered through ACCHSs and AMSs focus on providing holistic and culturally competent health care. |
| **Decolonisation** | Decolonisation refers to the process of undoing colonialism. While this can refer to the dismantling of colonial empires, existing cultures, practices, systems, attitudes and policies continue to perpetuate the philosophies underpinning colonialism. Decolonisation in this context is the process undertaken by systems and individuals to challenge dominant perspectives and restore Indigenous knowledges, cultures and ways. |
| **Cultural safety** | Where both healthcare providers and healthcare systems reflect on the impact of personal assumptions, biases, attitudes and prejudices on the quality of healthcare that is delivered to people. Cultural safety acknowledges that these perspectives arise from the individual’s cultural or social values. However, cultural safety also involves a process of self-reflection and awareness of these perspectives so that healthcare providers and organisations can address these assumptions and biases, and progress towards equity in the healthcare setting. |
